# Idiopathic adult intestinal intussusception: a rare cause of an acute surgical abdomen

**DOI:** 10.1093/jscr/rjaa542

**Published:** 2020-12-31

**Authors:** Eliot R Gange, Marco A Grieco, Scott D Myers, Timothy M Guenther

**Affiliations:** Department of Surgery, University of California Davis, Sacramento, CA 95817, USA; Department of Surgery, David Grant USAF Medical Center, CA 95433, USA; Department of Surgery, University of California Davis, Sacramento, CA 95817, USA; Department of Surgery, David Grant USAF Medical Center, CA 95433, USA; Department of Radiology, David Grant USAF Medical Center, CA 95433, USA; Department of Surgery, University of California Davis, Sacramento, CA 95817, USA; Department of Surgery, David Grant USAF Medical Center, CA 95433, USA

## Abstract

Intussusception is uncommon among adults. The condition, which is defined as a telescoping of a proximal portion of the small or large bowel into the lumen of an adjacent segment of bowel, is most commonly seen in children. Among pediatric cases, the majority is benign and treated non-operatively. However, in adults, intussusception is the result of pathologic and often malignant lead points in the majority of cases. This makes surgical resection and tissue diagnosis the only definitive treatment option. While the majority of adult intussusception cases involves a pathologic lead point, a small percentage is idiopathic, without an identifiable lead point. We present a 32-year-old man with acute on chronic abdominal pain and cross-sectional imaging that identified jejunal intussusception, which was confirmed in operating room and resected. Interestingly, no pathologic lead point was identified on pathologic review. We discuss our diagnostic approach, surgical decision making and final tissue diagnosis.

## INTRODUCTION

Intestinal intussusception refers to the phenomena whereby a segment of the intestine ‘telescopes’ into another segment secondary to peristaltic contraction of the intestine and a lead point [1, 2]. Four anatomic variants have been described including: (i) entero-enteric, involving only the small intestine, (ii) colo-colic, involving only the colon, (iii) ileo-colic, when terminal ileum prolapses into the ascending colon and (iv) ileo-cecal, when ileocecal valve is the lead point of the intussusception [3]. Intussusception is more common in pediatric patients with an estimated incidence of approximately 30 cases per 100 000 live births usually between ages 6 and 36 months [1]. In children, common presenting symptoms include cramping intermittent abdominal pain, bloody diarrhea and a palpable tender mass; however this triad is present in <50% of pediatric patients that present with intestinal intussusception [1]. In these younger patients, lead points causing intestinal intussusception are most commonly idiopathic, but benign etiologies such as lymphoid tissue proliferation can also occur. Because most pediatric cases of intussusception are ileo-colic, pneumatic or hydrostatic decompression of the intussusception can be successful in as high as 80% of cases with no need for resection of the involved segment of intestine [1, 3].

**Figure 1:**
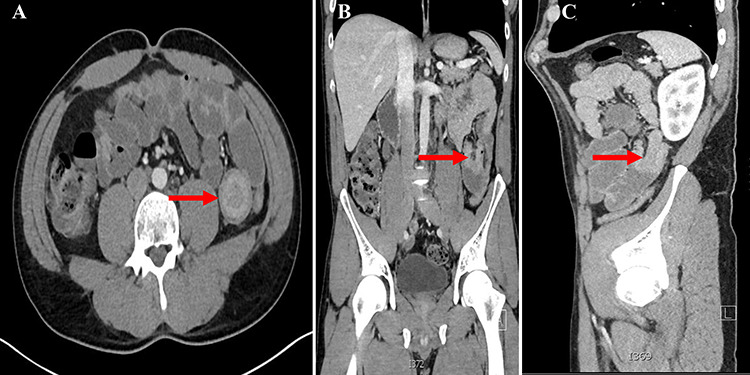
**A**. CT abdomen axial view showing the ‘Target Sign’ present within the small bowel; **B.** CT abdomen coronal view showing invagination of a proximal portion of small bowel into the adjacent section of bowel; **C**. CT abdomen sagittal view showing the aforementioned invagination of small bowel.

**Figure 2:**
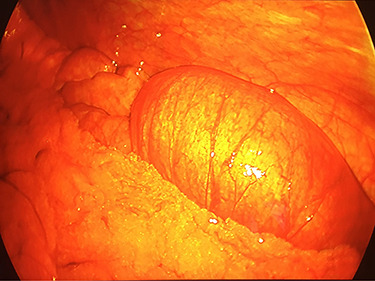
Intra-operative laparoscopic view of the intussuscepted small bowel prior to resection.

**Figure 3:**
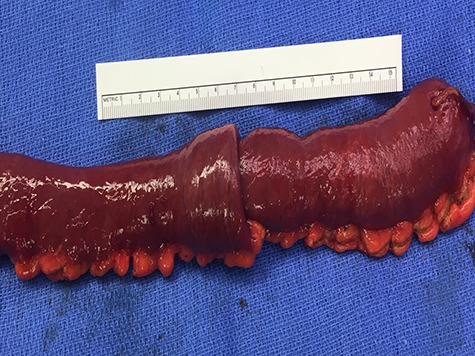
Gross resection specimen.

In contrast, adult onset intestinal intussusception is a rare cause of abdominal pain with an estimated incidence of 1–5% of all bowel obstructions. Lead points in this cohort are more commonly pathologic with etiologies such as carcinoma, polyps, Meckel’s diverticulum, or mesenteric cysts [4, 5]. For this reason, resection of the affected portion of bowel is typically recommended in adults with evidence of intussusception.

We report a 32-year-old man who presented with acute on chronic abdominal pain with cross sectional imaging that identified jejunal intussusception. This was confirmed in operating room and resected, however no pathologic lead point was identified on pathologic review.

## CASE REPORT

A 32-year-old otherwise healthy man presented to the emergency room with 4 h of severe cramping abdominal pain, nausea and bilious emesis. He described normal stools and his last bowel movement was just prior to presentation. The patient experienced a similar episode 2 years prior and a computerized tomography (CT) scan at the time identified jejunal intussusception. The patient underwent a diagnostic laparoscopy, which showed normal appearing bowel without evidence of intussusception. He denied any other surgeries and was unsure of any family history of inflammatory bowel disease as he was adopted.

On exam, the patient was afebrile and tachycardic to 110 beats/minute. He appeared in acute distress but was oriented to person, place and time. His abdomen was non-distended but was rigid with severe abdominal pain with palpation in all four quadrants. Laboratory evaluation identified a leukocytosis to 16.5 K/mm^3^ with normal serum electrolytes and liver function tests. A CT was obtained, which showed a 4-cm segment entero-enteric intussusception without obstruction or signs of ischemia with non-specific bowel thickening proximal to the intussusception (Fig. 1). Given peritonitis on exam, the patient was taken to the operating room where the entero-enteric intussusception was identified and resected in bloc (Figs 2 and 3) No masses were palpated in the surgical specimen.

The patient recovered well and was discharged on post-operative day 3. Pathologic review of the resected entero-enteric intestine showed intramural hemorrhage and surface changes consistent with early ischemia. No malignant tissue or other identifiable cause of the intussusception was observed. He has since been seen in clinic with near resolution of his chronic abdominal pain.

## DISCUSSION

Intussusception is a relatively rare diagnosis in the setting of severe abdominal pain and bowel obstruction in the adult surgical patient. In review of the literature, the majority of cases seem to follow a chronic and variable course of abdominal pain and symptoms of intermittent obstruction. This somewhat obscure clinical presentation can make timely diagnosis challenging and can delay uncovering a potential malignant cause chronic abdominal pain. Approximately 90% of intussusception cases in adults involve a pathologic lead point and of those, two-thirds are the result of either benign or malignant neoplasia [6]. Many studies report ~50% of pathologic lead points being of malignant etiology, the most common of which are adenocarcinoma, carcinoid tumors and leiomyosarcoma [6]. With the high probability of a pathologic lead point, surgical intervention is generally recommended in adults with intussusception, especially in those with signs of obstruction [7, 8]. As with any approach to the abdomen, the use of laparoscopic versus open technique should be based on the patient’s clinical status and the surgeon’s ability to perform the procedure safely and effectively.

The current data available for this disease process cannot yet fully elucidate its pathophysiology and clinical patterns. There is not yet sufficient evidence to suggest unifying characteristics shared among patients with idiopathic intussusception. Although idiopathic intussusception contributes to only a small percentage of bowel obstructions, understanding its pathophysiology and clinical presentation is an important aspect of surgical care.

In conclusion, adult idiopathic intussusception although rare, is deserving of additional investigation to further understand its causes and to facilitate efficient and effective surgical care.
